# The impact of digital health literacy on online learning engagement among undergraduate nursing students: the chain mediating roles of academic self-efficacy and future work self-salience

**DOI:** 10.1186/s12912-025-04235-x

**Published:** 2025-12-17

**Authors:** Yan Liu, Zhi-yuan Cheng, Jia Tao, Yu-qing Liang, Yue Zhang, Jie Wang, Zhou-tong Dai, Yi-ran Yue, Chun-rong Zhou, Li-li Chen, Wen-ting Xia, Dan Su

**Affiliations:** 1 School of Nursing, Anhui Medical University, 81 Meishan Road, Shushan District, Hefei, Anhui Province 230032 China; 2 The First Affiliated Hospital of Anhui Medical University, Hefei, Anhui China

**Keywords:** Health literacy, Education, distance, Self efficacy, Motivation, Students, Nursing

## Abstract

**Background:**

Amid the rapid expansion of digital nursing education, digital health literacy is considered key to enhancing students’ engagement in online learning. However, the underlying mechanism of this relationship remains unclear. This study examines the relationship between digital health literacy and online learning engagement, while also examining the roles of academic self-efficacy and future work self-salience in this process.

**Methods:**

A cross-sectional study was conducted between February and March 2024, involving 518 undergraduate nursing students from two medical universities in Anhui Province, China. Data were collected using the Digital Health Literacy Scale, the Academic Self-Efficacy Scale, the Future Work Self-Salience Scale, and the Online Learning Engagement Scale.

**Results:**

The mean score for online learning engagement among undergraduate nursing students was 53.83 (8.16). Digital health literacy exerted a significant total effect on online learning engagement (β = 0.781). This total effect comprised both a direct effect (β = 0.400) and a significant total indirect effect (β = 0.381) mediated by academic self-efficacy and future work self-salience. Notably, academic self-efficacy and future work self-salience played an important chain-mediating role in this relationship (β = 0.065), accounting for 8.32% of the total effect.

**Conclusions:**

Digital health literacy is a significant positive predictor of online learning engagement. It enhances students’ academic self-efficacy, which in turn clarifies their future work self-salience, ultimately promoting higher online learning engagement. Therefore, nursing educators should not only strengthen students’ digital health literacy but also foster their academic confidence and career foresight. Such strategies are crucial for improving the quality of digital nursing education and student learning outcomes.

**Clinical trail number:**

Not applicable.

## Introduction

### Background

The rapid advancement of the digital era necessitates continuous learning to adapt to new technologies [1]. This trend is precipitating a digital transformation in higher nursing education, with online learning emerging as a core component [2]. While this shift offers unprecedented opportunities for information access, it also poses significant challenges. Notably, online learning environments, in contrast to traditional face-to-face instruction, can diminish student engagement [3], potentially compromising learning outcomes. In a practice-oriented discipline such as nursing, this decline is particularly concerning, as it threatens to undermine students’ clinical competence and professional identity [4]. Therefore, identifying strategies to enhance online learning engagement is imperative for the academic success and career readiness of undergraduate nursing students, who are at a pivotal stage of their professional identity development.

Online learning engagement refers to the investment of cognitive, emotional, and behavioral resources by students during the online learning process [5]. It is widely recognized as a key predictor of academic success and learning satisfaction across multiple disciplines [6, 7]. For instance, research in the United States indicates that highly engaged students achieve more profound knowledge mastery and exhibit greater persistence [8]. Therefore, in the context of digital nursing education, fostering high engagement is not merely an academic objective but a professional imperative. It is essential for enabling students to effectively leverage digital resources in mastering complex clinical concepts, thereby laying a solid foundation for their professional careers.

Among the factors influencing online learning engagement, digital health literacy is emerging as a significant area of interest [9]. Distinct from general digital literacy [10], digital health literacy is defined as the ability to seek, understand, evaluate, and apply health-related information from digital sources to inform health decisions [11]. For nursing students, digital health literacy is not merely an auxiliary skill but a core competency essential for their future professional practice. It cultivates critical thinking skills in information processing and health decision-making [12]. Research has already demonstrated that general digital literacy is positively associated with students’ overall academic performance [13]. Furthermore, a study focusing on nursing students revealed that the more specialized digital health literacy is a key predictor of their health-promoting behaviors [14]. This positive influence may similarly extend to their online learning engagement.

Beyond skill-based literacy, intrinsic psychological factors are critical influences on online learning behavior [15]. Among these, academic self-efficacy, a concept rooted in Bandura’s social cognitive theory, is particularly prominent. Academic self-efficacy refers to an individual’s belief in their capability to complete specific academic tasks successfully [16]. In online learning environments that lack immediate supervision and support, the role of academic self-efficacy as an internal factor is especially crucial [17]. This intrinsic belief shapes students’ learning attitudes, effort expenditure, and resilience when facing challenges, thereby influencing their learning outcomes [16]. Congruently, students with higher academic self-efficacy are more proactive in their learning, more persistent in overcoming difficulties, and exhibit greater online learning engagement [18]. This confidence is particularly beneficial for nursing students, as it not only bolsters their engagement with theoretical material but also enhances their participation in online practical simulations and activities [19]. Thus, fostering this confidence is crucial for ensuring that nursing students can translate their online learning into real-world professional readiness.

Students’ perceptions of their future career also shape their current learning engagement [20]. Future work self-salience refers to the psychological clarity and accessibility of an individual’s expected future professional role, goals, and developmental path [21]. A clear, well-defined vision of a future career can infuse current learning with a sense of purpose, thereby enhancing intrinsic motivation [22]. Conversely, the demanding and uncertain nature of the nursing profession may diminish the perceived value of current academic work and reduce student initiative [23]. For example, a study of nearly 700 nursing students found that when online education inadequately addresses practical skills, it can erode students’ confidence and professional clarity, consequently lowering their learning engagement [24]. This suggests that enhancing domain-specific competencies may foster online learning engagement by solidifying students’ future professional identity.

While previous studies have separately examined the roles of digital literacy, self-efficacy, and career goals [25, 26], several notable limitations persist. First, the majority of research has focused on general digital literacy [27], overlooking the more critical domain-specific digital health literacy for nursing students. Second, few studies have integrated these skill-based, cognitive, and motivational factors into a comprehensive model to elucidate the pathway through which digital skills influence online learning engagement. Finally, despite the rapid digital transformation of nursing education in mainland China [28], integrative research within this specific context remains scarce. Specifically, the collective influence of these factors on students’ online learning engagement has not been systematically investigated. Therefore, this study aims to examine the relationships among digital health literacy, academic self-efficacy, future work self-salience, and online learning engagement. Furthermore, it seeks to elucidate the underlying mechanisms through which digital health literacy influences online learning engagement among undergraduate nursing students.

### Theoretical background and research hypotheses

**Fig. 1:**
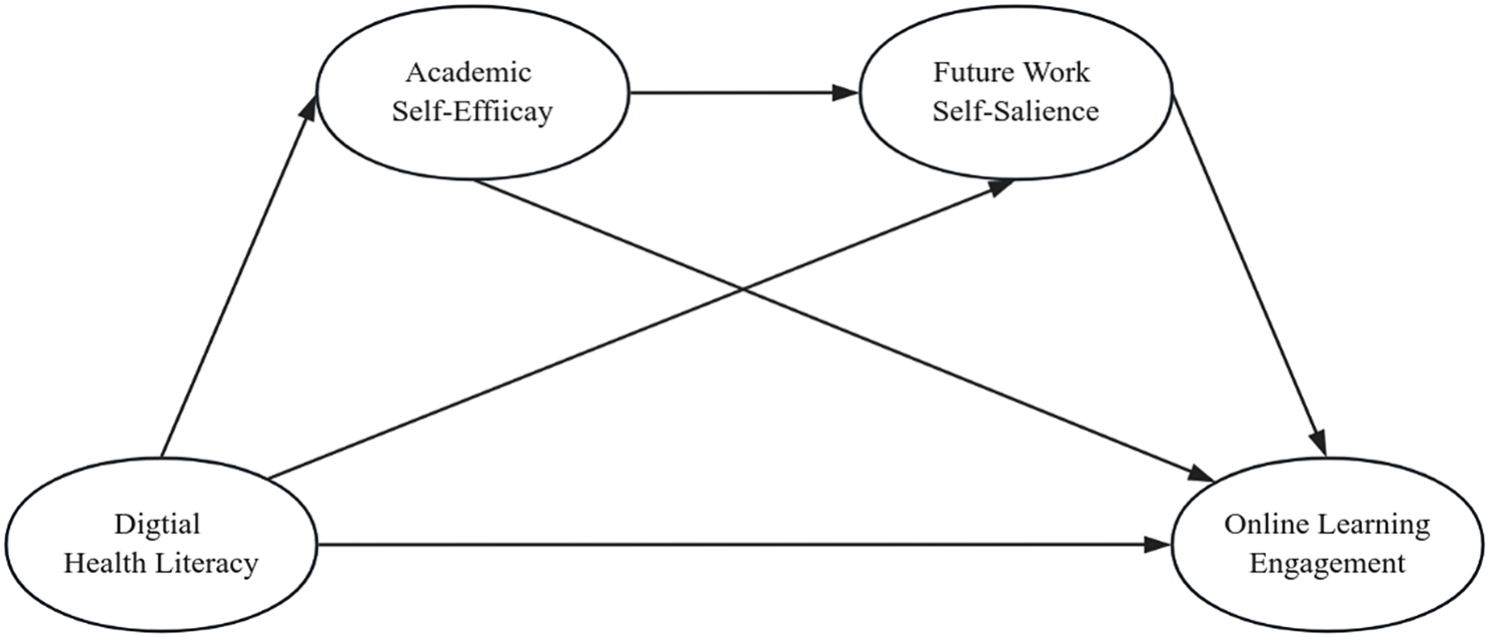
Hypothesized model of the interrelationships among digital health literacy, academic self-efficacy, future work self-salience, and online learning engagement

This study is guided by Social Cognitive Career Theory (SCCT). This theory posits that learning experiences shape key cognitive variables, such as self-efficacy and outcome expectations, thereby influencing behavior. These cognitive factors, in turn, shape personal goals and subsequent behaviors [29]. In this study, digital health literacy is regarded as a key learning experience highly relevant to the nursing profession. It shapes students’ beliefs and goals by fostering two core psychological variables: academic self-efficacy and future work self-salience. These variables are valuable internal resources for nursing students. Particularly under intense academic and clinical pressures, they help sustain learning engagement and ultimately influence online learning behaviors [30]. They subsequently influence the final behavioural outcome, namely online learning engagement. Therefore, drawing on SCCT, this study investigates the serial mediating roles of academic self-efficacy and future work self-salience in the relationship between digital health literacy and online learning engagement among undergraduate nursing students. Accordingly, the hypothesized model is presented in Fig. 1.

#### Hypothesis 1

**(H1)**: Digital health literacy will be positively associated with online learning engagement.

A substantial body of research has established that general digital and information literacy is a key factor in the online learning engagement and academic performance of university students [31]. For instance, a study of university students in the United Arab Emirates found that digital competence was a critical predictor of their online course engagement and success [32]. Similarly, research in the United States has confirmed that higher information literacy is strongly associated with more active academic participation [33]. When this literacy is integrated with a specific professional domain, its effect becomes more pronounced. For nursing students, a higher level of digital health literacy can enhance their efficiency in using online resources and mitigate frustration from information overload or invalid information, thereby enabling them to construct their knowledge systems more effectively [34]. Therefore, we hypothesized that digital health literacy, as a more domain-specific competency, would have a direct, significant positive impact on nursing students’ online learning engagement.

#### Hypothesis 2

**(H2)**: Academic self-efficacy will mediate the relationship between digital health literacy and online learning engagement.

According to SCCT, successful learning experiences strengthen an individual’s self-efficacy [29]. This enhanced academic belief, in turn, can motivate students to adopt more active learning strategies, such as setting higher goals and exerting greater effort, ultimately leading to higher levels of online learning engagement [33]. A study of British undergraduates found that self-efficacy is positively correlated with digital health literacy [35]. Moreover, academic self-efficacy has been identified as a significant predictor of learning engagement [36]. Therefore, we hypothesized that digital health literacy promotes online learning engagement by enhancing academic self-efficacy.

#### Hypothesis 3

**(H3)**: Future work self-salience will mediate the relationship between digital health literacy and online learning engagement.

According to SCCT, personal goals are direct precursors to behavior [29]. Specifically, a clear and meaningful future goal provides a strong sense of purpose for current learning tasks, which in turn stimulates online learning engagement [37]. When nursing students perceive online learning content as a necessary step toward their career aspirations, their motivation is enhanced, leading to more active engagement [38]. Moreover, formulating clear career goals requires relevant information and skills. Research has confirmed that digital literacy is crucial for academic and career development in the digital age [39]. Therefore, we hypothesized that digital health literacy promotes online learning engagement by enhancing future work self-salience.

#### Hypothesis 4

**(H4)**: Academic self-efficacy and future work self-salience will sequentially mediate the relationship between digital health literacy and online learning engagement.

Prior research has shown that academic self-efficacy positively predicts an individual’s motivation for career and academic pursuits [40], and indirectly influences online learning engagement through its impact on career goals [41]. This finding is consistent with SCCT, which posits that efficacy beliefs are a primary driver of an individual’s career goals [29]. Students with high academic self-efficacy, believing in their ability to succeed, are more likely to explore and commit to specific career paths, thereby developing a clearer and more salient future work self [42]. In turn, a clear future career goal provides powerful intrinsic motivation for current online learning, prompting students to engage more actively in their studies to realize these long-term aspirations [43]. Therefore, we hypothesized that academic self-efficacy and future work self-salience sequentially mediate the relationship between digital health literacy and online learning engagement.

## Methods

### Study design

A cross-sectional study design was adopted. Data were collected from a convenience sample of participants via an anonymous online questionnaire. This study was reported in accordance with the Strengthening the Reporting of Observational Studies in Epidemiology (STROBE) statement.

### Setting and participants

Data were collected between February 10 and March 28, 2025 at two major medical universities in Anhui Province, China. These institutions were selected for their extensive use of blended-learning tools and resources, including learning management systems (e.g., SuperStar Learning, Rain Classroom), videoconferencing tools for instruction (e.g., DingTalk, VooV Meeting), and Massive Open Online Courses (MOOCs). The universities recruit students from across the nation, resulting in a geographically diverse participant pool. Participants were required to meet the following inclusion criteria: (1) be a full-time undergraduate nursing student; (2) provide informed consent; (3) be capable of independently understanding and completing the questionnaire; and (4) have used an online learning platform at least once within the preceding 12 months. The exclusion criteria were: (1) any cognitive or communication impairment that would hinder questionnaire comprehension and completion; (2) concurrent participation in another research study on a similar topic.

### Sample size

The sample size was calculated using the formula n = (U_α_σ/δ)^2^ for cross-sectional studies [44]. In this study, α = 0.05 and δ = 0.50 were taken, and the standard deviation of the total online learning engagement score available through the pre-survey before the formal survey was 5.35. Given the possibility of invalid questionnaires, the sample size was increased by 20%, resulting in a sample of 528 cases.

### Measures

#### Demographic characteristics

A questionnaire, designed explicitly for this study, was administered to collect demographic information from the participants. The data gathered included age, gender, residential area, student leadership experience, the number of personal online devices, average daily time spent on online learning, satisfaction with the online learning platform, and any prior digital technology training.

#### Digital health literacy

Digital health literacy was measured using the Chinese version of the Digital Health Information Literacy (DHIL) scale. This scale was initially developed by van der Vaart et al. [45] and later adapted by Zhao [46]. The scale comprises two sections: a self-assessment and an objective assessment. The self-assessment section includes 21 items designed to measure perceived abilities across seven dimensions (three items per dimension): operational skills, web navigation skills, information searching, reliability appraisal, relevance appraisal, adding web content, and privacy protection. Items are rated on a 5-point Likert scale, ranging from 0 (Very difficult) to 4 (Very easy). The total score for this section ranges from 0 to 84, with higher scores indicating greater perceived digital health literacy. The objective assessment section consists of seven items designed to measure actual skills, with one item per dimension. For these items, correct answers were scored as 1, while incorrect or I don’t know responses were scored as 0. In this study, the Cronbach’s α for the scale was 0.962.

#### Academic self-efficacy

Academic self-efficacy was assessed using the Chinese version of the Academic Self-Efficacy Scale (ASES). This instrument was originally developed by Pintrich and DeGroot [47] and subsequently adapted by Liang [48], whose validation study reported good reliability (Cronbach’s α = 0.817). The scale comprises 22 items that measure two dimensions: self-efficacy for learning ability (11 items) and self-efficacy for learning behavior (11 items). Items were rated on a 5-point Likert scale, ranging from 1 (Completely disagree) to 5 (Completely agree). Total scores range from 22 to 110, with higher scores indicating a greater level of academic self-efficacy. In this study, the Cronbach’s α for the scale was 0.944.

#### Future work self-salience

Future work self-salience was measured using the Chinese version of the Future Work Self-Salience (FWSS) scale. This scale was initially developed by Strauss et al. [21] and later adapted by Guan [49], whose validation study reported good reliability (Cronbach’s α = 0.920). The scale is unidimensional and comprises four items. These items were rated on a 5-point Likert scale, ranging from 1 (Strongly disagree) to 5 (Strongly agree). Total scores range from 4 to 20, with higher scores indicating greater future work self-salience. In this study, the Cronbach’s α for the scale was 0.894.

#### Online learning engagement

Online learning engagement was assessed using the Chinese version of the Online Learning Engagement Scale (OLE). This instrument was initially developed by Li et al. [50] and subsequently revised by Zhu [51], whose validation study reported good reliability (Cronbach’s α = 0.888). The scale comprises 15 items that assess three dimensions: behavioral engagement (6 items), emotional engagement (4 items), and cognitive engagement (5 items). Items were rated on a 5-point Likert scale, ranging from 1 (Completely disagree) to 5 (Completely agree). Total scores range from 15 to 75, with higher scores indicating greater online learning engagement. In this study, the Cronbach’s α for the scale was 0.934.

### Data collection

Data were collected from February 10 to March 28, 2025. Before the formal survey, a pilot study was conducted with 10 undergraduate nursing students to evaluate the questionnaire’s clarity, comprehensibility, and completion time. The instrument was revised based on the feedback received. For the formal survey, the anonymous questionnaire was administered using the Wenjuanxing online platform (www.wjx.cn). A link to the study was disseminated to eligible participants via the WeChat application. An introductory statement outlined the study’s purpose and assured participants of anonymity to protect their privacy. To ensure data quality, all questions were set to mandatory, and submissions were limited to one per IP address, preventing missing data and duplicate entries. A total of 542 completed questionnaires were received. After data cleaning, 24 responses were excluded: 10 due to uniform responses (i.e., selecting the same option for all items) and 14 due to abnormally short completion times compared to the pilot study average. This process yielded a final valid sample of 518 participants, yielding an effective response rate of 95.57%.

### Data analysis

Data analysis was performed using SPSS 23.0 and AMOS 24.0. Initially, Harman’s single-factor test was conducted to assess for common method bias (CMB); bias was considered problematic if the first factor accounted for 50% or more of the total variance [52]. Subsequently, descriptive statistics were calculated. Categorical variables were summarized as frequencies and percentages. Continuous variables were first evaluated for normality using Kolmogorov-Smirnov tests and Q-Q plots. As the data approximated a normal distribution, they were described using means and standard deviations. To compare online learning engagement scores across demographic groups, independent-samples t-tests and one-way ANOVA were used. Pearson correlation analysis was then used to examine the bivariate relationships between digital health literacy, academic self-efficacy, future work self-salience, and online learning engagement. Finally, structural equation modeling (SEM) was performed using AMOS 24.0 to test the hypothesized chain-mediation model. Model parameters were estimated via maximum likelihood, and the model was refined using modification indices. The goodness-of-fit of the model was evaluated against established criteria: the chi-square/degrees of freedom ratio (χ²/df) < 5, root mean square error of approximation (RMSEA) ≤ 0.08, comparative fit index (CFI) ≥ 0.90, goodness-of-fit index (GFI) ≥ 0.90, and normed fit index (NFI) ≥ 0.90. The significance of the indirect effects was tested using a bootstrapping procedure with 5000 resamples. An effect was considered statistically significant if its 95% bias-corrected confidence interval (CI) did not include zero [53]. For all statistical tests, the significance level was set at *p* < 0.05 (two-tailed).

### Ethical considerations

This study was approved by the Ethics Committee of Anhui Medical University (Approval No. 83230280), and all procedures were performed in accordance with the ethical principles of the Declaration of Helsinki. Before participation, written informed consent was obtained from all individuals. Participants were fully informed of the study’s purpose, procedures, and their right to withdraw at any time without penalty. Furthermore, they were assured of the strict confidentiality of their personal data and the anonymity of their responses.

## Results

### Sociodemographic information of participants

The study included 518 undergraduate nursing students. The mean age of participants was 20.18 (1.04), and the majority were female (*n* = 391, 75.5%). The most commonly used online learning tools were Superstar Learning (92.9%), MOOCs (70.1%), DingTalk or VooV Meeting (54.3%), and Rain Classroom (50.0%). Regarding satisfaction with these platforms, a majority of students (58.3%) reported a moderate level of satisfaction. Group comparisons revealed that online learning engagement scores did not differ significantly by gender, age, or the number of online devices owned (all *p* > 0.05). However, statistically significant differences in engagement scores were found for family residence (t = 2.12, *p* = 0.035), holding a student cadre position (t = 3.37, *p* < 0.001), daily online learning time (t = -3.23, *p* = 0.001), receipt of digital technology training (t = 3.69, *p* < 0.001), and overall platform satisfaction (F = 21.11, *p* < 0.001) (Table 1).

**Table 1:** Online learning engagement scores of undergraduate nursing students by demographic characteristics (*N* = 518)

Variable	*N* (%)	Score (Mean ± SD)	t/F	*p*
Gender				
Male	127 (24.52)	53.70 ± 9.01	-0.20	0.842
Female	391 (75.48)	53.87 ± 7.87		
Age				
≤ 20 years	344 (66.41)	53.72 ± 8.09	-0.42	0.672
> 20 years	174 (33.59)	54.04 ± 8.31		
Family Residence				
Urban	228 (44.02)	54.68 ± 8.06	2.12	0.035
Rural	290 (55.98)	53.16 ± 8.19		
Student Leadership Experience				
Yes	265 (51.16)	55.00 ± 8.00	3.37	<0.001
No	253 (48.84)	52.60 ± 8.15		
Number of Online Devices				
1	133 (25.68)	52.39 ± 8.62	2.79	0.062
2	252 (48.65)	54.31 ± 7.88		
3 or more	133 (25.67)	54.35 ± 8.10		
Daily Online Learning Time				
≤ 2 h	384 (74.13)	53.15 ± 8.02	-3.23	0.001
> 2 h	134 (25.87)	55.77 ± 8.27		
Received Digital Tech Training?				
Yes	386 (74.52)	54.59 ± 8.24	3.69	<0.001
No	132 (25.48)	51.59 ± 7.52		
Platform Satisfaction				
Unsatisfied	38 (7.33)	52.13 ± 11.04	21.11	<0.001
Average	302 (58.30)	52.21 ± 7.54		
Satisfied	178 (34.47)	56.93 ± 7.59		

### Descriptive statistics and correlation analysis

The mean score for online learning engagement was 53.83 (8.16), indicating a moderate level of engagement. The correlation analysis revealed significant positive intercorrelations among all study variables (*p* < 0.01). Future work self-salience showing high positive correlations with both digital health literacy (*r* = 0.725, *p* < 0.01) and online learning engagement (*r* = 0.700, *p* < 0.01). Furthermore, online learning engagement showed strong positive relationships with both digital health literacy (*r* = 0.563, *p* < 0.01) and academic self-efficacy (*r* = 0.561, *p* < 0.01) (Table 2).

**Table 2:** Pearson correlations among digital health literacy, academic self-efficacy, future work self-salience, and online learning engagement (*N* = 518)

Variable	Mean (SD)	DHL	ASE	FWSS	OLE
DHL	57.66 (11.79)	1			
ASE	75.66 (11.87))	0.523**	1		
FWSS	13.59 (2.67)	0.725**	0.675**	1	
OLE	53.83 (8.16)	0.563**	0.561**	0.700**	1

Note: DHL, digital health literacy; ASE, academic self-efficacy; FWSS, future work self-salience; OLE, online learning engagement

**p < 0.01

### Structural equation model and mediation analysis

**Fig. 2:**
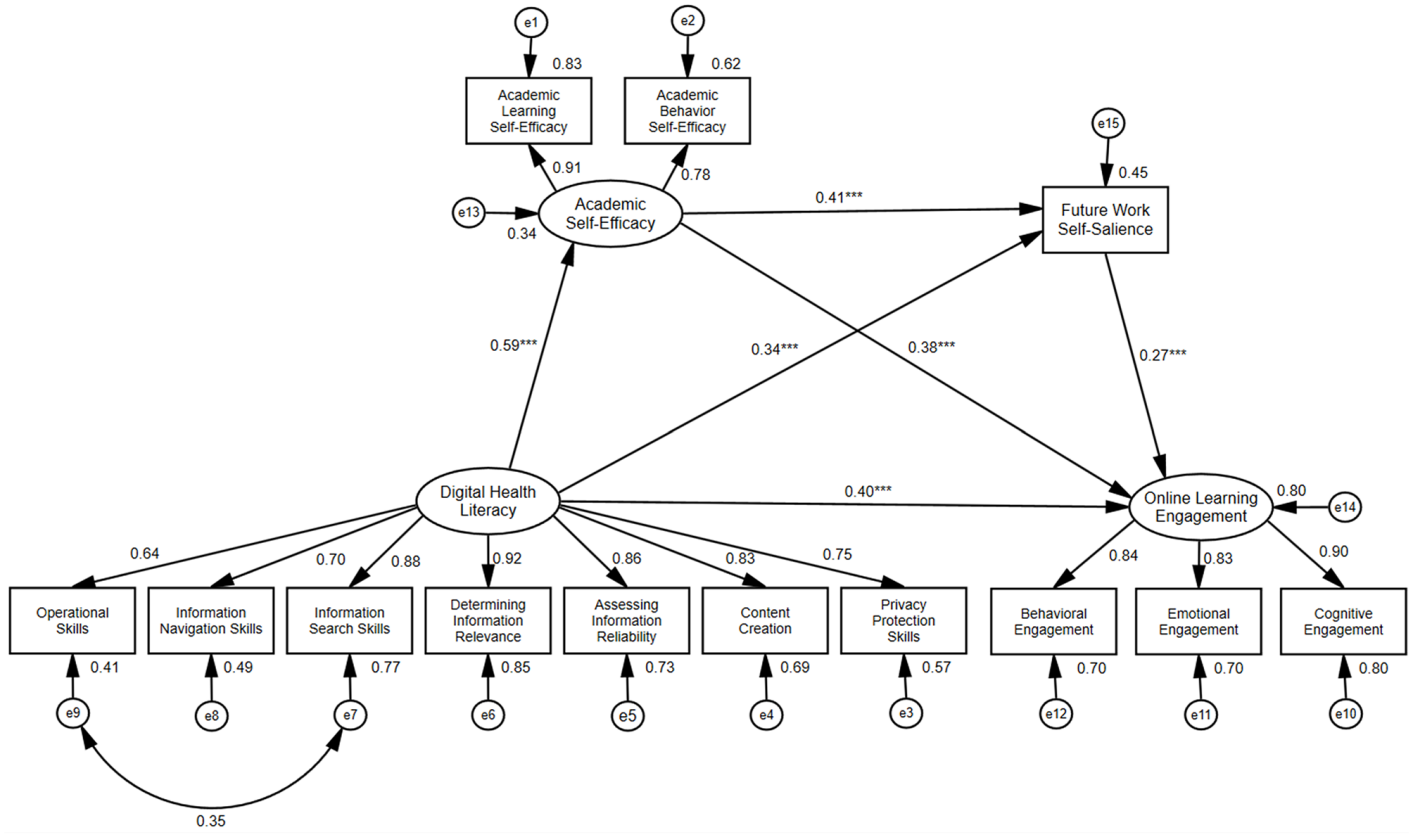
Final model of the impact of digital health literacy, academic self-efficacy, and future work self-salience on online learning engagement. **P < 0.01, ***P < 0.001

The mediation analysis (Fig. 2) indicated a significant total effect of digital health literacy on online learning engagement (β = 0.781, *p* < 0.001). The total indirect effect, mediated jointly by academic self-efficacy and future work self-salience, was also significant (β = 0.381, 95% CI [0.309, 0.458], *p* < 0.001), accounting for 48.78% of the total effect. A decomposition of the indirect effects (Table 3) revealed three significant mediation pathways. First, the path mediated solely by academic self-efficacy was significant (β = 0.224, 95% CI [0.129, 0.346], *p* < 0.001), explaining 28.68% of the total effect. Second, the path mediated by future work self-salience alone was also significant (β = 0.092, 95% CI [0.042, 0.174], *p* < 0.001), explaining 11.78% of the total effect. Finally, the sequential mediation path through both academic self-efficacy and future work self-salience was significant (β = 0.065, 95% CI [0.050, 0.112], *p* < 0.001), accounting for an additional 8.32% of the total effect.

**Table 3:** Bootstrap analysis of chain mediating effects of academic Self-Efficacy and future work Self-salience between digital health literacy and online learning engagement (*N* = 518)

Paths	β	BootSE	Bootstrap 95% CI(bias corrected)		*p*	Proportion of total effect(%)
			Lower	Upper		
DHL→ASE→OLE	0.224	0.057	0.129	0.346	0.001	28.68
DHL→FWSS→OLE	0.092	0.033	0.042	0.174	0.000	11.78
DHL→ASE→FWSS→OLE	0.065	0.015	0.050	0.112	0.000	8.32
Indirect Effect(DHL→OLE)	0.381	0.038	0.309	0.458	0.001	48.78
Direct Effect (DHL→OLE)	0.400	0.049	0.304	0.491	0.000	51.22
Total Effect	0.781	0.027	0.719	0.826	0.000	100

Note: β, regression coefficient; SE, standard error; CI, confidence interval. DHL, digital health literacy; ASE, academic self-efficacy; FWSS, future work self-salience; OLE, online learning engagement

## Discussion

The findings of this study indicate that digital health literacy, academic self-efficacy, future work self-salience, and online learning engagement were significantly and positively associated among undergraduate nursing students. Furthermore, academic self-efficacy and future work self-salience were found to sequentially mediate the relationship between digital health literacy and online learning engagement. Collectively, these results support all of the study’s proposed hypotheses.

### Direct impact of digital health literacy on online learning engagement

The research results confirmed H1, indicating that digital health literacy has a significant direct positive impact on undergraduate nursing students’ online learning engagement. This finding is consistent with the literature that emphasises the critical role of digital competence for success in online learning environments [46]. A study in Hungary also found that university students’ digital competence was a key predictor of their online learning success [54]. In the context of nursing, digital health literacy, as a core competency, transcends mere technical skills. It enables students to effectively acquire, discern, integrate, and apply knowledge to solve professional problems. Students with higher digital health literacy can utilise digital resources more fluently, thereby reducing the cognitive load and frustration caused by technical barriers or information overload [55]. Notably, our findings also revealed a discrepancy: while students performed well on basic information retrieval tasks, they were less proficient in specific, context-based applications, such as navigating a simulated hospital website. This highlights a critical gap between general digital skills and applied, domain-specific digital health literacy. Therefore, nursing education should undergo a transformation in teaching. It is no longer sufficient to teach the technical operation of digital tools. Instead, to effectively enhance online learning engagement, curricula must be intentionally designed to cultivate students’ ability to critically evaluate and apply information in complex, authentic health-related contexts. This approach is essential for bridging the identified gap and preparing students for lifelong learning.

### Mediating role of academic self-efficacy

The findings confirmed H2, demonstrating that academic self-efficacy plays a significant mediating role in the relationship between digital health literacy and online learning engagement. This result aligns with key tenets of both Bandura’s self-efficacy theory and Social Cognitive Career Theory, which posit that mastery experiences are a primary source of efficacy beliefs [56]. For nursing students, successfully applying digital skills to complete professional tasks constitutes a critical mastery experience. This not only builds their confidence in using digital tools but also enhances their broader academic self-efficacy to meet the demands of online education [57]. This enhanced self-efficacy is particularly critical in online learning environments, which require a high degree of autonomy and self-regulation [58]. It fosters a resilient belief in their ability to overcome learning challenges. This link is supported by a study from Getenet et al. [59], which reported that higher efficacy beliefs were associated with stronger cognitive engagement and resilience. Conversely, students with low digital health literacy may struggle to find relevant information or question the validity of their sources, leading to repeated negative online experiences [60], thereby lowering academic self-efficacy. This can manifest as task avoidance, procrastination, or superficial engagement. Therefore, academic self-efficacy is not merely a correlated factor; it is a core psychological mechanism that translates digital competence into sustained, positive learning behaviors. In the evolving landscape of digital nursing education, curricula should move beyond mere skill acquisition to deliberately cultivate academic self-efficacy, which underpins students’ ability to thrive in digital learning environments.

### Mediating role of future work self-salience

The findings confirmed H3, revealing that future work self-salience significantly mediates the relationship between digital health literacy and online learning engagement. This result aligns with the career development literature, which emphasizes that establishing a clear link between current learning activities and future career expectations enhances student motivation and engagement [61]. In the context of this study, mastery of digital health literacy, as a professionally relevant competency, enhances nursing students’ confidence in and identification with their future professional roles [34], thereby strengthening their future work self-salience. According to SCCT, a salient future work self is closely linked to an individual’s career goals and positive outcome expectations [29]. When students possess a clear vision of their professional future, they perceive current academic tasks as having greater value and meaning. This value-driven intrinsic motivation, in turn, prompts them to engage more deeply with online coursework, which they view as a direct pathway to achieving their long-term goals [62]. Therefore, our findings suggest that integrating digital health literacy into nursing education extends beyond mere skill acquisition; it also helps students clarify their professional identity and goals, which in turn drives their current online learning engagement. In practice, digital health literacy initiatives should explicitly connect online coursework to future professional roles to enhance student engagement.

### Chain mediating role of academic self-efficacy and future work self-salience

A key contribution of this study is the confirmation of H4, which established the chain mediating role of academic self-efficacy and future work self-salience in the relationship between digital health literacy and online learning engagement. This finding underscores that a single determinant does not govern online learning engagement but instead results from a complex interplay of multiple psychological factors [63]. The significance of this psychological mechanism is particularly pronounced in the high-pressure Chinese educational context. In an environment characterized by widespread academic and employment pressure, learning motivation stemming from intrinsic beliefs and clear goals is more stable and sustainable than that driven by external evaluations [64]. Corroborating this, research on Chinese university students has identified self-efficacy and clear academic goals as core protective factors for sustaining learning motivation amidst academic challenges [65]. Specifically, academic self-efficacy played the role of transforming external learning experiences into internal motivation. This conviction encourages students to adopt active coping strategies rather than avoidance behaviors when facing online learning challenges [66], thereby preparing them psychologically for subsequent career exploration. Building on this foundation of self-efficacy, future work self-salience then bridges this internal belief to long-term career development. By imbuing current learning tasks with a sense of value and purpose, it enables students to perceive online coursework as an essential pathway toward achieving their personal and professional goals [21]. Consequently, our findings suggest that synergistic interventions targeting both academic self-efficacy and future work self-salience can more effectively leverage the positive impact of digital health literacy on online learning engagement. This offers a novel direction for optimizing online learning strategies for undergraduate nursing students.

## Limitations

The current study has several limitations that need to be addressed in future research. First, the cross-sectional design, while identifying significant associations, cannot establish definitive causality. Future research should employ longitudinal or experimental designs to more rigorously examine the causal pathways and dynamic interplay between digital health literacy, self-efficacy, future work self-salience, and online learning engagement over time. Second, although the study revealed the impact pathway of digital health literacy on online learning engagement and verified the mediating roles of academic self-efficacy and future work self-salience, the mechanisms underlying online learning engagement may involve additional factors. The mechanisms of interaction among related elements require further exploration. Finally, the study was conducted within a specific Chinese educational context and was limited to nursing students. This may affect the universality of our conclusions, as the weighting and role of factors such as academic pressure and career expectations can vary across cultures and scholarly disciplines. Therefore, cross-cultural comparative studies are needed to validate the model’s robustness and explore potential differences in the salience of its pathways. Furthermore, interventions based on these findings should be adapted to incorporate context-specific elements to enhance their cross-cultural effectiveness and applicability.

## Conclusion

The results revealed that digital health literacy predicts online learning engagement not only directly but also indirectly through the chain-mediating pathway of academic self-efficacy and future work self-salience. Therefore, nursing education must extend beyond mere technical skill training. A dedicated focus on simultaneously enhancing students’ academic self-efficacy, clarifying their career paths, and solidifying their domain-specific digital competence is crucial for translating current academic success into long-term career readiness.

## Data Availability

The data in this research can be obtained from the corresponding author on reasonable request.

## Abbreviations

SCCT: Social Cognitive Career Theory
DHIL: Digital Health Information Literacy Scale
ASES: Academic Self-Efficacy Scale
FWSS: Future Work Self Salience Scale
OLE: Online Learning Engagement Scale
ANOVA: Analysis of Variance
CMB: Common Method Bias
SPSS: Statistical Package for the Social Sciences
AMOS: Analysis of Moment Structures
SEM: Structural Equation Modeling
MOOCs: Massive Open Online Courses
STROBE: Strengthening the Reporting of Observational Studies in Epidemiology
CI: Confidence Interval
SE: Standard Error
